# Phenology-dependent cold exposure and thermal performance of *Ostrinia nubilalis* ecotypes

**DOI:** 10.1186/s12862-020-1598-6

**Published:** 2020-03-06

**Authors:** Crista B. Wadsworth, Yuta Okada, Erik B. Dopman

**Affiliations:** 1 Department of Biology, Tufts University, 200 Boston Ave, Suite 4700, Medford, MA 02155 USA; 2 Current Affiliation: Rochester Institute of Technology, Thomas H. Gosnell School of Life Sciences, 85 Lomb Memorial Drive, Rochester, NY 14623 USA

**Keywords:** Thermal tolerance, Seasonal timing, Phenology, Cold-hardiness, Climate adaptation, Diapause, Adaptation by time

## Abstract

**Background:**

Understanding adaptation involves establishing connections between selective agents and beneficial population responses. However, relatively little attention has been paid to seasonal adaptation, in part, because it requires complex and integrative knowledge about seasonally fluctuating environmental factors, the effects of variable phenology on exposure to those factors, and evidence for temporal specialization. In the European corn borer moth, *Ostrinia nubilalis*, sympatric pheromone strains exploit the same host plant (*Zea mays*) but may genetically differ in phenology and be reproductively “isolated by time.” Z strain populations in eastern North America have been shown to have a prolonged larval diapause and produce one annual mating flight (July), whereas E strain populations complete an earlier (June) and a later (August) mating flight by shortening diapause duration. Here, we find evidence consistent with seasonal “adaptation by time” between these ecotypes.

**Results:**

We use 12 years of field observation of adult seasonal abundance to estimate phenology of ecotype life cycles and to quantify life-stage specific climatic conditions. We find that the observed reduction of diapause duration in the E strain leads their non-diapausing, active life stages to experience a ~ 4 °C colder environment compared to the equivalent life stages in the Z strain. For a representative pair of populations under controlled laboratory conditions, we compare life-stage specific cold tolerance and find non-diapausing, active life stages in the E strain have as much as a 60% greater capacity to survive rapid cold shock. Enhanced cold hardiness appears unrelated to life-stage specific changes in the temperature at which tissues freeze.

**Conclusions:**

Our results suggest that isolation by time and adaptation by time may both contribute to population divergence, and they argue for expanded study in this species of allochronic populations in nature experiencing the full spectrum of seasonal environments. Cyclical selective pressures are inherent properties of seasonal habitats. Diverse fluctuating selective agents across each year (temperature, predation, competition, precipitation, etc.) may therefore be underappreciated drivers of biological diversity.

## Background

Our understanding of ecological adaptation has been greatly increased by studies of species evolving to spatially distinct areas or to different resources in the same geographic region. Important studies include those emphasizing insects on different host plants [1], plants on different soils [2], birds feeding on different seeds [3], fish living in different aquatic habitats [4], and snails living at different tidal elevations [5]. However, a significant gap in knowledge concerns how divergent natural selection might drive adaptive divergence when populations evolve to environments that are temporally rather than spatially distinct.

Adaptation to temporal environments is a potentially powerful form of ecological adaptation because it can drive phenotypic divergence and speciation in two key ways. The same area or resource can be exploited at different times (e.g., spring versus summer), and different areas or resources often differ in seasonal timing (e.g., northern versus southern habitats; different host plants). Consequently, organisms can experience divergent selection on phenology to synchronize with different exploitable temporal environments (e.g., [1, 6–9]. When differences in phenology are genetic and influence reproductive timing, assortative mating and temporally restricted gene flow can evolve (“isolation by time”) [10, 11]).

A second opportunity for adaptive divergence occurs because selection may often vary with phenology. Organisms living in seasonal habitats may often experience fluctuating selection pressure due to seasonal changes in abiotic and biotic factors such as temperature, precipitation, natural enemies, or competitors (e.g., [12–14]. Due to these seasonal ecological contrasts, selection during one temporal environment could to lead to rise in mean fitness as trait means approach a new optima, while potentially also bringing about a decline in mean fitness with respect to an earlier or later temporal environment. Evolution in distinct temporal environments associated with phenology shifts can thus promote additional adaptive trait divergence and reductions in gene flow (“adaptation by time”) [10].

Allochronic ecological adaptation therefore consists of two phenomena that may jointly drive phenotypic divergence and speciation. Although partial temporal isolation is frequently observed between closely related taxa [15], the potential for adaptation to distinct time periods remains controversial. Evidence suggests that the magnitude and stability of allochrony can vary. For example, plasticity in timing or variable strength of temporal isolation between populations can allow for “dispersal”, gene flow, and an erosion of seasonal adaptation [15–17]. It is also possible for seasonal traits to be environmentally influenced or condition dependent, or even for a single trait value to enable optimal exploitation across temporal environments [18–20]. Empirical studies of allochronic adaptation are rare, in part, because they require connecting phenology across the entire life cycle with life-stage specific performance and in seasonal environments that can change from year-to-year. If divergent natural selection in temporal environments does play an important role during ecological speciation, allochronic populations should experience some sort of ecological contrast and performance differences should be evident when allochronic populations experience a different temporal environment (e.g., [13, 21]. Here, we evaluate evidence for these predictions in the European corn borer moth (*Ostrinia nubilalis*).

The European corn borer (ECB) moth is a plant-feeding insect native to Europe, and was imported accidentally to North America in the early 1900’s [22]. The larval (borer) stage damages plants by destroying internal plant tissue as they feed. Larvae have been found on ~ 200 species of hosts [23, 24], but corn (*Zea mays*) is the most commonly exploited resource in North America [25, 26]. Multiple phenotypes reproductively isolate populations, but the strongest involve heritable differences in mate selection and timing of overwintering larval diapause [27]. Across regions of central and western Europe, and also eastern North America, sympatric populations differ in male preference for divergent female pheromone blends, resulting in partial behavioral isolation between “E” and “Z” pheromone strains [17, 22, 28]. Across native and introduced ranges, pheromone strains feeding and overwintering within corn hosts can also differ in larval diapause timing, leading to partial temporal reproductive isolation where they co-occur [17, 29, 30]. Due to this shift in larval diapause timing, univoltine Z adults mate mid-season (late July) and have only a single generation of offspring (univoltine ecotype), whereas bivoltine E adults mate earlier (early June) and then produce a second-generation mating flight at the end of the season (mid-August) (bivoltine ecotype) [31, 32]. Fewer than 30% of bivoltine E (BE) and univoltine Z (UZ) mating flights may be synchronous at sympatric locations, which is estimated to cause a 60–85% reduction in gene flow relative to synchronic populations [27]. Despite these barriers, evidence for hybridization and genome-wide introgression occurs in nature, except at the loci underlying timing and pheromone communication traits and at a large chromosomal rearrangement on the Z (sex) chromosome [17, 33–35].

**Fig. 1:**
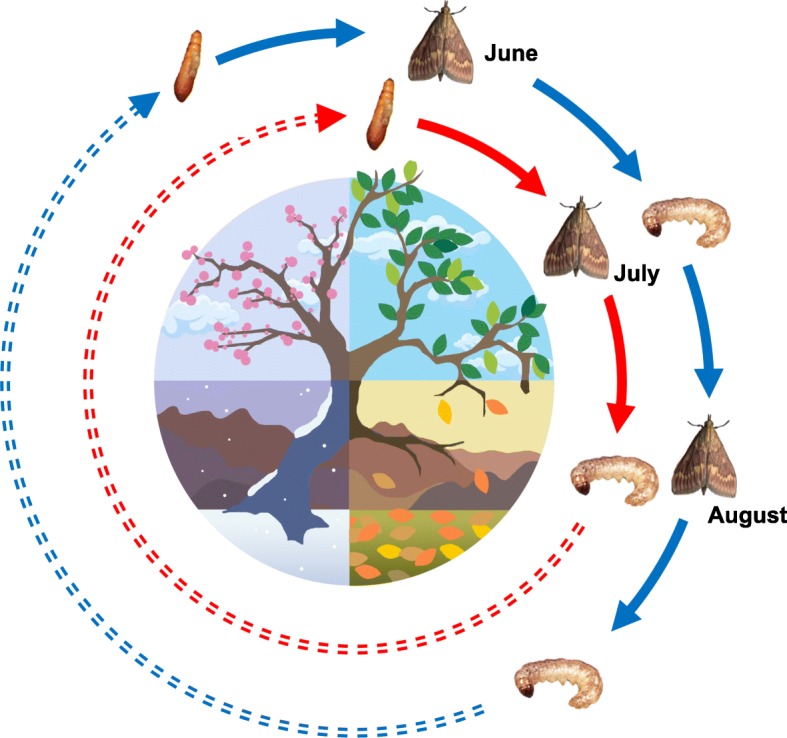
Genetically-based shifts in seasonal timing potentiate seasonal adaptation. Two partially temporally isolated pheromone strains of European corn borer moth (*Ostrinia nubilalis*) exist in eastern North America. Z strain populations (red) have a prolonged larval diapause (dashed line) and produce one mating flight (July), whereas E strain populations (blue) complete an early (June) and late (August) flight by shortening diapause. Due to a combination of seasonal fluctuations in temperature and reduced diapause duration, direct-developing E strain pupae and larvae could be subject to more extreme thermal environments than the same life stages in the Z strain. In this study, we test for thermal adaptation by time in corn borer strains by life stage

Although ECB moth populations may show evidence of isolation by time, adaptation by time has not been studied. A potentially important source of selection between allochronic bivoltine E and univoltine Z populations is temperature. Diapausing life stages in insects are commonly cold hardy and like many insects [36, 37], overwintering hardiness in *Ostrinia* during diapause is mediated by glycerol accumulation and freeze avoidance [38–40]. In contrast to diapausing life stages, direct-developing life stages in insects are often vulnerable to cold stress [41, 42]. Compared to univoltine Z populations, bivoltine E populations can fit two generations per year by ending larval diapause earlier in the spring and also by entering diapause later in the fall [30, 43, 44]. As a consequence of earlier release and later entry into diapause, non-diapausing active life stages preceding and following diapause in bivoltine E populations may experience colder seasonal environments than equivalent life stages in univoltine Z populations (Fig. 1). Therefore, colder temperatures may have selected for enhanced cold tolerance in bivoltine E individuals, especially for sensitive life stages preceding and following diapause (direct-developing larvae and pupae). Alternatively, corn borers could have a large range of thermal tolerance that is not costly to maintain, differences in temperature could be minor resulting in weak selection, or gene flow between the bivoltine E and univoltine Z populations could erode trait divergence.

We report an initial study of adaptation by time that focuses on moths from central New York. Using 12 years of field observations, we estimate phenology of bivoltine E and univoltine Z life cycles in this part of the species range and the seasonal temperatures experienced across life stages. We then experimentally compare life-stage specific cold tolerance between a well-studied pair of allochronic E and Z populations that have been previously characterized for seasonal timing and other ecologically relevant traits [17, 27, 30, 34, 45, 46]. Finally, we examine possible physiological mechanisms underlying trait variation.

## Results

### Life stage phenology

To understand how variation in phenology relates to seasonal variation in temperature, we first estimated seasonal timing of bivoltine E and univoltine Z moths. We estimated life-stage specific phenology using weekly abundance data for adult mating flights from Farmington, New York, originally collected by the New York State Cooperative Extension Service. For predictions of adult male flight periods, the year 2009 for the Z strain and 2010 for the E strain were omitted due to low sample sizes (*N* < 20 adult males). In the remaining years, average adult male flight periods from 1999 to 2010 fell between June 4–17 for the first flight of the E strain, July 31–August 21 for the second flight of the E strain, and June 29 - July 30 for the only flight of the Z strain. From these adult distributions, we predicted the phenology of other life stages using the number of degree days (DD) required for development [47] (Supplementary Figure 1).

**Fig. 2:**
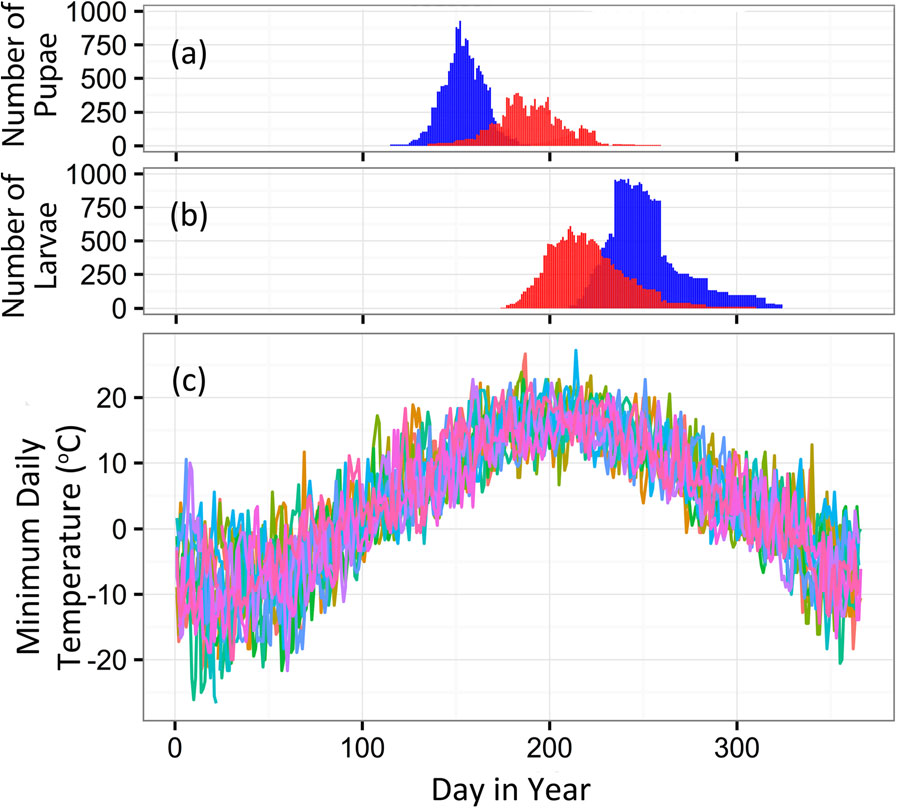
Seasonal temperature and life-history timing of univoltine and bivoltine populations. Estimated seasonal timing of (**a**) direct developing pupae and (**b**) first through fourth instar larvae of bivoltine E (blue) and univoltine Z (red) populations for years 1999–2010. **c** Minimum daily temperature at Geneva Research Farm, NY for years 1999–2010, with each year represented by a unique color

As temperatures begin to rise in spring, direct developing pupae in the E strain were predicted to occur earlier in the year (Fig. 2a). Average first occurrence of E strain pupae was May 14 ± 3.5 days, and average last occurrence of pupae was June 19 ± 2.9 days. For the same years, the average first occurrence of Z strain pupae was June 17 ± 5.7 days, and average last occurrence was July 30 ± 6.2 days.

As temperatures begin to drop in autumn, direct developing larvae in the E strain were predicted to occur later in the year (Fig. 2b). For this analysis, a range of direct developing larval instars (first occurrence of the 1st instar to the first occurrence of the 4th instar) of the univoltine Z strain were compared to the equivalent life stages for the second generation of bivoltine E strain. In 2000, 2003, and 2004 for the E strain, and in 2003 for the Z strain, there were not enough degree days within a season for the entire predicted larval cohort to reach the 4th instar before winter. Therefore, we used the last day above 10 °C as the end cutoff to define the temporal and thermal habitat in these instances. These may have been high mortality years in which not all individuals reached the proper stage (5th larval instar) to enter diapause successfully. For the second generation of the E strain, we predicted the average first occurrence of 1st instar larvae as August 10 ± 3.20 days, and the first occurrence of fourth instar larvae as October 13 ± 8.3 days (Fig. 2b). For the Z strain, the average first occurrence of 1st instar larvae was July 11 ± 3.8 days, and the first occurrence of fourth instar larvae was September 3 ± 8.8 days.

### Cold exposure

To determine how evolution of diapause phenology relates to life-stage specific exposure to seasonal temperature, thermal ranges were characterized across the life cycle of each strain using daily climate data mined from National Oceanic and Atmospheric Administration (NOAA) archives. Based on predicted phenology, pupal and larval life stages in the E strain were exposed to a colder thermal environment than equivalent life stages in the Z strain (Fig. 2c).

**Fig. 3:**
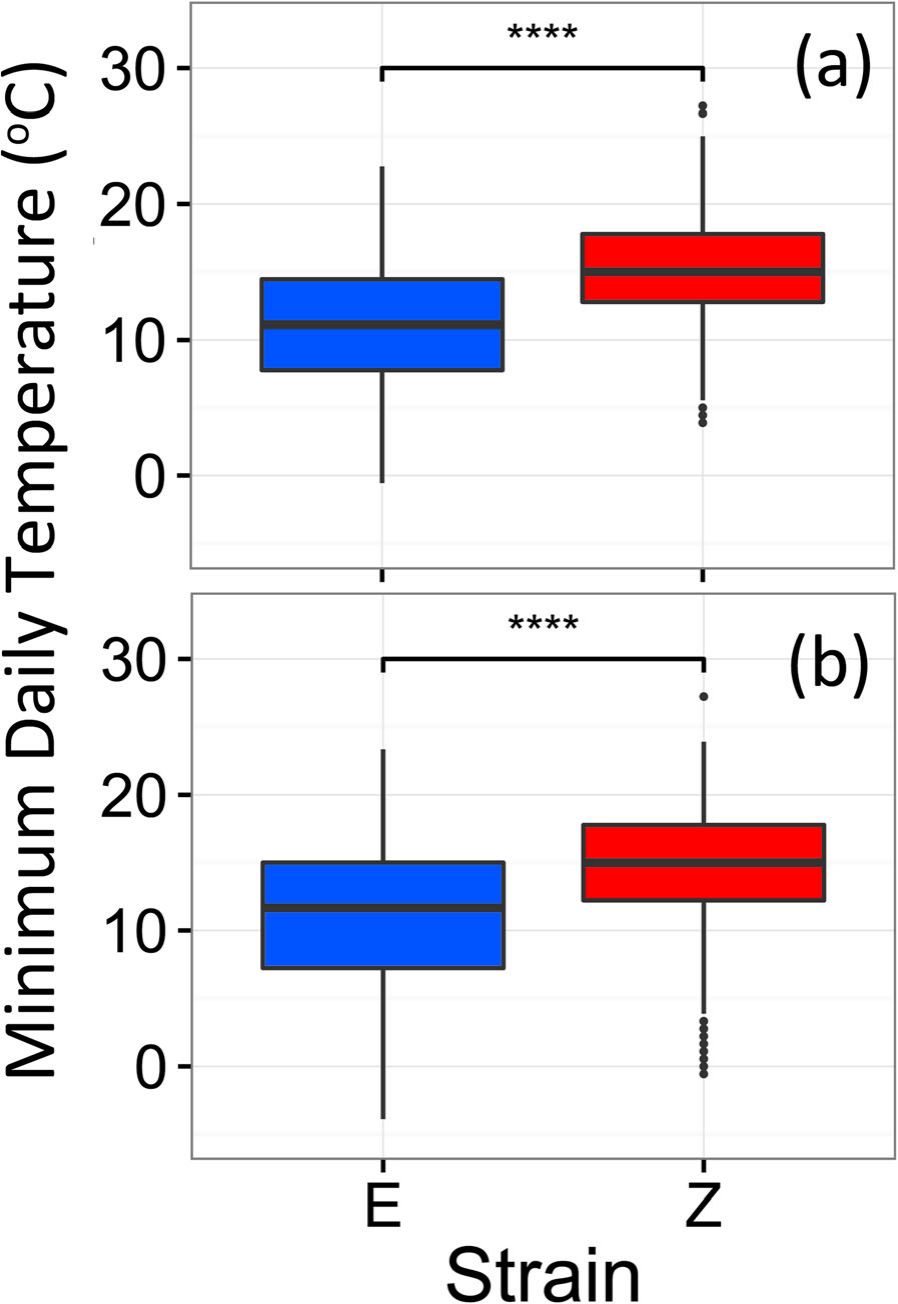
Estimated exposure of sensitive life stages to cold temperature extremes. Minimum daily temperatures reported from 1999 to 2010 for the predicted temporal niches of (**a**) bivoltine E and univoltine Z pupae (Wilcox Rank Sum, W = 141,100, *p* < 0.0001), and (**b**) first through fourth instar larvae of E (second-generation) and Z (Wilcox Rank Sum, W = 296,600, *p* < 0.0001)

Over the 12 years investigated, daily maximum and minimum ambient air temperatures for the predicted pupal ranges were collected to define the thermal environments that each strain typically experiences. The maximum daily temperatures during the pupal stage ranged from 6.7 °C to 35.6 °C (mean = 22.2 °C, median = 22.2 °C) for the E strain and 10.6 °C to 35.0 °C (mean = 25.5 °C, median = 25.8 °C) for the Z strain (Supplementary Figure 2). Over the same period, the minimum daily temperatures during the pupal stage ranged from − 0.6 °C to 22.8 °C (mean = 11.1 °C, median = 11.1 °C) for the E strain and 3.9 °C to 27.2 °C for the Z strain (mean = 15.1 °C, median = 15.0 °C) (Fig. 3). Differences in thermal environments of the first occurring pupal stages between strains were highly statistically significant for both the maximum daily temperatures (Wilcox Rank Sum, W = 154,390, *p* < 0.0001) and minimum daily temperatures (Wilcox Rank Sum, W = 141,100, *p* < 0.0001).

The maximum daily temperatures during the 1st through the 4th instars ranged from − 0.6 °C to 36.1 °C (mean = 21.3 °C, median = 22.8 °C) for the E strain and 2.2 °C to 36.1 °C (mean = 25.0 °C, median = 25.6 °C) for the Z strain (Supplementary Figure 2). The minimum daily temperatures ranged from − 5.6 °C to 23.3 °C (mean = 10.8 °C, median = 11.7 °C) for the E strain and − 0.6 °C to 27.2 °C (mean = 14.6 °C, median = 15.0 °C) for the Z strain (Fig. 3). As in the pupal stages, the thermal environments were highly statistically significantly different for both the maximum daily temperatures (Wilcox Rank Sum, W = 283,840, *p* < 0.0001) and minimum daily temperatures (Wilcox Rank Sum, W = 296,600, *p* < 0.0001).

### Lethal temperature

**Fig. 4:**
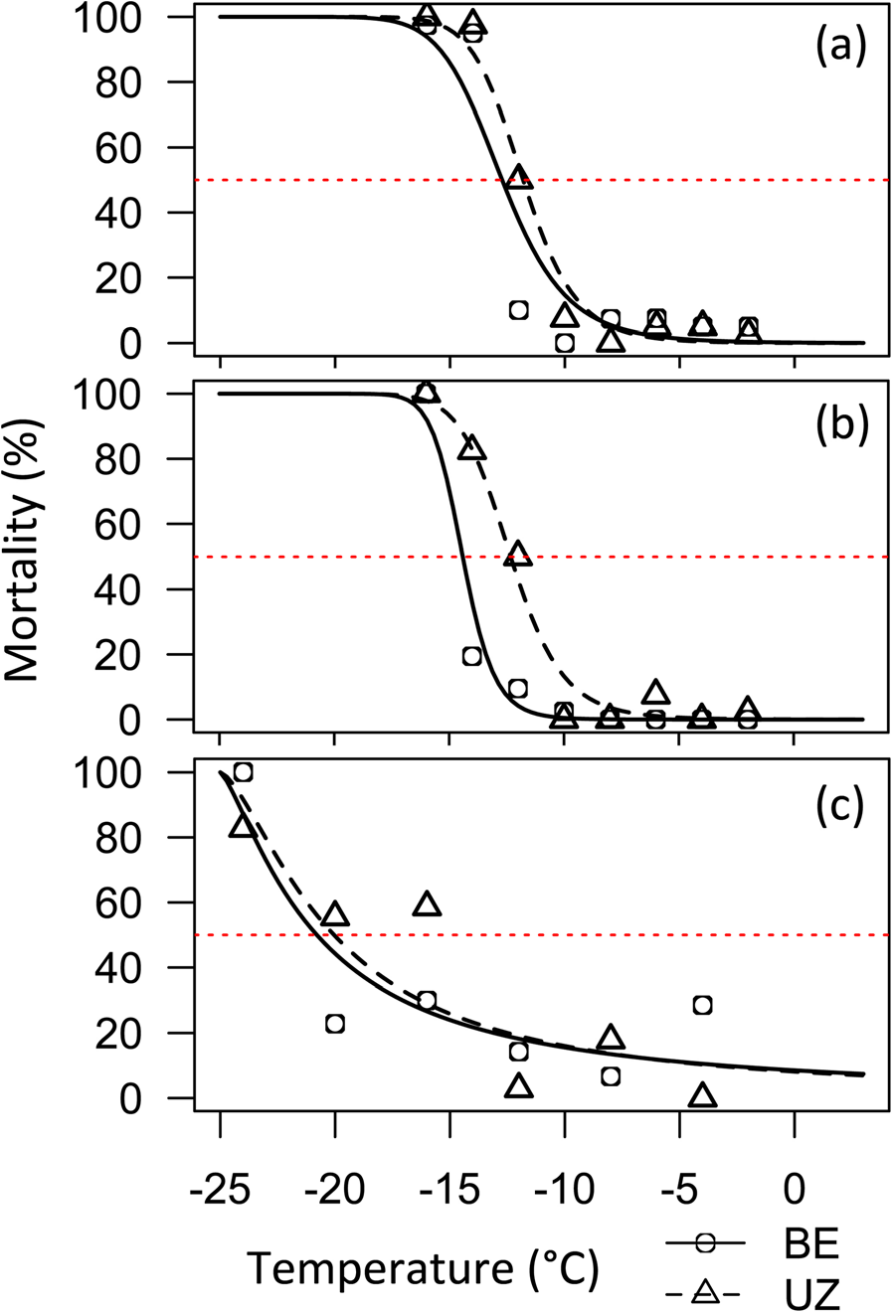
Differences in lethal temperature between one and two generation populations. Log-logistic function fit to mortality data to predict LT50s. Panel **a** shows fits for the pupal life stage, panel **b** shows fits for the direct developing larval life stage, and panel **c** shows fits for cold-acclimated diapause larvae. The bivoltine E (BE) strain is represented by circles and a solid line, while the univoltine Z (UZ) strain is represented by triangles and a dashed line. Red dotted line shows 50% mortality

To determine if differences in thermal habitats as a result of phenology shifts might have selected for evolution of lower thermal limits, corn borers strains were subjected to cold exposures at a variety of sub-zero temperatures across life stages. Lethal temperatures (LT) and supercooling points (SCP) were measured for the direct developing life stage that follows diapause (pupa), a representative direct developing life stage preceding diapause (non-diapausing 5th instar larva), and the diapausing life stage (cold-acclimated diapausing 5th instar larva). Ten days after subzero temperature exposure, direct developing individuals had either died, had pupated in larval groups, or eclosed in pupal groups. Similarly, cold-acclimated diapause larvae had either died, pupated, or remained as viable larvae that could move vigorously when disturbed. Estimates for the lethal temperature for 50% mortality in each population were lower in E strain borers across all life stages compared to the Z strain (Table 1; Fig. 4a-c). However, LT50s were significantly different between strains in direct developing larval and pupal stages, but not in the cold-acclimated diapause stage (Table 2). Within the E strain, LT50 estimates were significantly different between all life stages, with the LT50 estimate the lowest in the cold-acclimated diapause larvae, and the highest in the pupal group (Tables 1 and 2; Fig. 4). The Z strain showed a different pattern where both the direct developing life stages had significantly higher LT50s than the cold-acclimated diapause group, but were not significantly different from one another (Tables 1 and 2; Fig. 4).

**Table 1:**
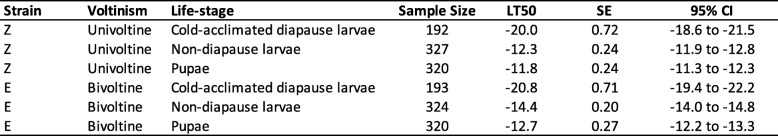
Lethal temperature 50 (LT50) estimates for corn borer strains by life-stage

**Table 2:**
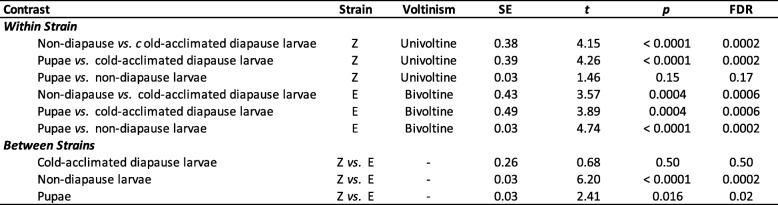
Results of lethal temperature 50 (LT50) ratio tests

**Fig. 5:**
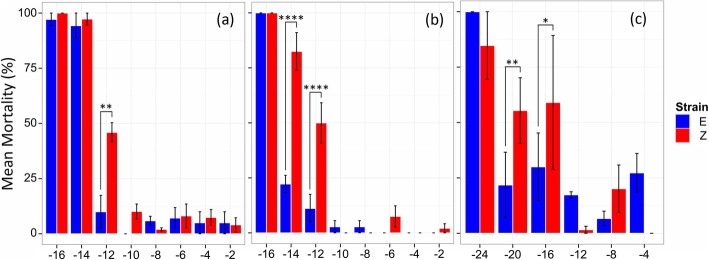
Differences in mortality at subzero temperatures between one and two generation population. **a** Pupae, **b** direct developing 5th instar larvae, and **c** cold-acclimated diapausing larvae. Bars represent the mean mortality in each group ± SE. Significance between strains at various subzero temperatures was determined using two GLMs followed post-hoc by Tukey’s HSD where * = *p* < 0.05, ** = *p* < 0.01, and **** = *p* < 0.0001

Consistent with LT50 estimates, E strain corn borers had higher survival at subzero temperatures than Z strain corn borers (Fig. 5a-c). For direct developing life stages, the main effects of temperature, strain, and life stage were significant, as were all two-way interactions (Table 3). The E strain had significantly better survival than the Z strain at − 12 °C in the direct developing larval (E strain mortality = 11.1%, Z strain mortality = 50%, Tukey’s HSD, z = − 4.52, *p* < 0.0001) and pupal (E strain mortality = 10%, Z strain mortality = 45.9%, Tukey’s HSD, z = − 3.58, *p* = 0.0019) life stages. E strain corn borers also had better survival than Z strain borers at the − 14 °C treatment in the direct developing larval stage (E strain mortality = 22.2%, Z strain mortality = 82.5%, Tukey’s HSD, z = − 5.03, *p* < 0.0001). For cold-acclimated diapause larvae, only temperature and the interaction between strain and temperature were significant (Table 3). Between strains, E strain cold-acclimated corn borers survived better at the − 16 °C (E strain mortality = 30%, Z strain mortality = 59%, Tukey’s HSD, z = − 2.17, *p* = 0.029) and − 20 °C (E strain mortality = 21.8%, Z strain mortality = 55.4%, Tukey’s HSD, z = − 2.75, *p* = 0.006) temperature exposures. At the lowest common temperature measured across all treatment groups (− 16 °C), cold-acclimated diapause larvae had significantly better survival than direct developing larvae for both the E (Fisher’s Exact test, *p* < 0.0001) and Z strains (Fisher’s Exact test, *p* < 0.0001).

**Table 3:**
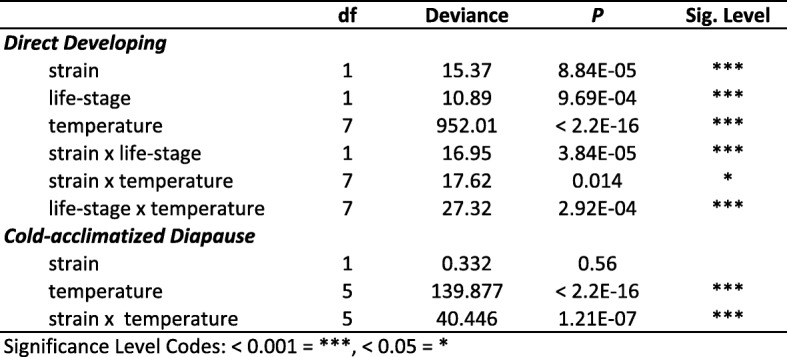
Results of generalized linear models testing the effect of temperature dose, strain, and life-stage on mortality after subzero temperature exposures

Significance Level Codes: < 0.05 = ^*^

### Supercooling point

**Fig. 6:**
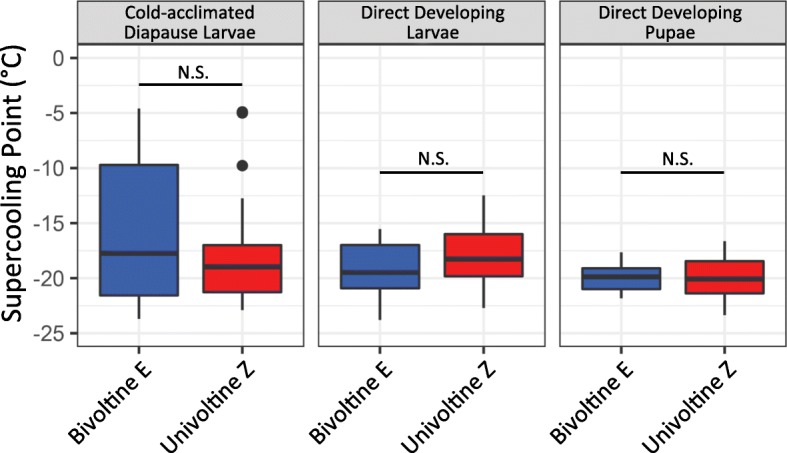
No significant differences in supercooling point between strains within life stage. Z (red) and E (blue) corn borer strains separated by life stage. For cold-acclimated diapause larvae, direct developing larvae, and direct developing pupae there were no significant differences in SCPs between strains within life stages

We found no significant difference in SCP between strains (Table 4 and Table 5; Fig. 6). Life stage was the only significant explanatory factor for SCP (Table 4). Cold-acclimated diapause larvae had a significantly higher SCP than non-diapause larvae (Tukey’s HSD, *p* = 0.04) and pupae (Tukey’s HSD, *p* = 0.00003). Direct developing life stages were not significantly different from one another (Tukey’s HSD, *p* = 0.10). The E strain cold-acclimated diapausing group had a significantly higher SCP than E strain direct developing larvae (Tukey’s HSD, *p* = 0.01), E strain pupae (Tukey’s HSD, *p* = 0.0006), and Z strain pupae (Tukey’s HSD, *p* = 0.0005) (Supplementary Figure 3). While both strains in the cold-acclimated diapause groups had higher SCPs than direct developing life stages, and a similar range from ~ − 5 °C to ~ − 23 °C, E strain corn borers had more individuals with higher SCPs, although there was no significant difference between groups (Tukey’s HSD, *p* = 0.33) (Supplementary Figure 3).

**Table 4:**
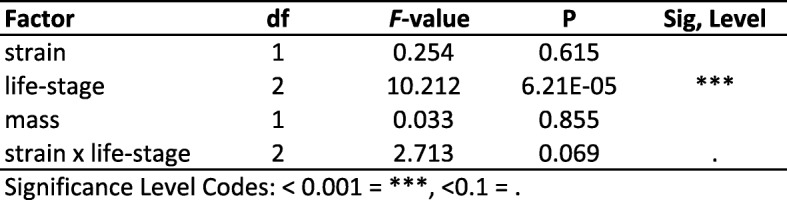
Results of ANCOVA testing the effects of strain, life-strange, and mass on supercooling point (C)

Significance Level Codes: < 0.0001 = ^***^, < 0.1=

**Table 5:**
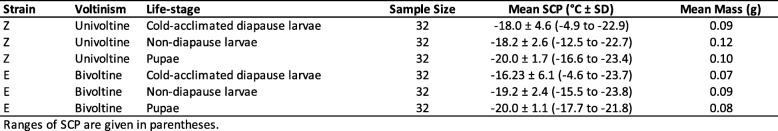
Summary of supercooling points (°C) for all life-stages by strain

Ranges of SCP are given in parentheses

## Discussion

We detected evidence for an ecological contrast between temporal environments of allochronic populations, and a performance difference when populations experience different temporal environments. Compared to univoltine Z populations, bivoltine E populations emerge from diapause 20–30 days earlier in the spring due to genetic differences in the timing of diapause termination [30, 43, 46] and they also enter diapause 20–30 days later in the fall due in part from genetic differences in the timing of diapause initiation [44]. Our results based on field observation support the presence of a difference in temperature associated with these phenology shifts. In central NY, earlier-emerging E strain pupae are predicted to experience minimum temperatures that are an average of 4 °C colder than Z strain pupae, which emerge approximately 1 month later (mid-May vs. mid-June) (Fig. 3a). Phenology change also exposes a range of second-generation E strain larvae to minimum temperatures that are 4 °C colder on average, compared to the same larval life stages in the single generation of Z strain borers (e.g., during early-September in the Z strain vs. mid-October in the E strain) (Fig. 3b).

Our results further suggest an association between the capacity to survive cold temperature and phenology shifts into colder seasonal environments. Under our experimental conditions, earlier-emerging E strain pupae survived cold treatment better than the same life stage in the Z strain, as indicated by a ~ 36% increase in cold-shock survivorship (− 12 °C) and a 1 °C shift in the lower thermal limit (Table 2; Fig. 4; Fig. 5a). Similarly, later-occurring larvae of the E strain had a greater capacity to tolerate cold compared to Z strain larvae, as indicated an increase in survival by ~ 40–60% upon cold exposure (− 12 °C, − 14 °C) and a 2 °C shift in lethal temperature (Table 2; Fig. 4; Fig. 5b). Populations were raised in a common garden for multiple generations; therefore, genetic rather than plastic or maternal effects are likely accounting for survival differences. The ability to cope with rapid decreases in temperature is thought to contribute to survival during seasonal cold snaps and day to night fluctuations in temperature [19]. Enhanced cold tolerance in the bivoltine E strain could provide a large fitness advantage in nature for E strain corn borers colonizing earlier and later temporal windows where extreme daily or seasonal temperature fluctuations approach the lower thermal limit for survival.

Divergence in thermal tolerance in other species is often associated with populations exploiting spatially distinct areas or different resources in the same geographic region (e.g., [1, 48, 49]; reviewed by [50]). Here however, divergent resource adaptation is unlikely because all ECB individuals were taken directly from the same overwintering microhabitat within sweet corn and there is no evidence of intra-host segregation between strains. Potential adaptation to geographic differences in climate cannot be fully excluded because our experimental populations are derived from sites separated by approximately 100 km. However, spatially varying selection is expected to be weak because sites do not significantly differ in monthly temperature (Supplementary Table 1).

Although observed patterns of performance and evolutionary pressure are consistent with predictions of adaptation by time, multiple directions for further study exist. More precise measurements of the thermal condition experienced by phenologically shifted populations could be made by quantifying seasonal temperature within whole corn stalks (during the growing season) and within corn stubble (overwinter), rather than using ambient air temperature. Cut corn stubble is open to the ambient air and is presumably less insulating to larval and pupal ECB than whole living plant tissue. Nevertheless, corn’s insulating properties are currently unknown, and the evolutionary pressure experienced by ECB populations could differ from that inferred from air temperature (e.g., due to maintenance of higher temperature or prevention of rapid temperature change) [51]. It is also clear that rapid cold shock response in controlled laboratory conditions will not reveal the effects of daily and seasonal temperature change on growth and development. Follow up studies of performance are needed in the field or using conditions that more closely mimic the ECB’s natural environment. Finally, additional allochronic population pairs from across the historic (Europe) and introduced (USA) range of this species will need to be explored before broad conclusions about thermal tolerance and its contribution to ecological speciation can be made in this system.

Correlations between seasonal timing and seasonal tolerance suggests that fluctuating natural selection in distinct temporal environments can contribute to the spread of coordinated seasonal adaptations, despite any homogenizing influence of gene flow and recombination [21, 52, 53]. Although cold tolerance was studied in populations from different sites, both were acquired from locations where bivoltine E and univoltine Z populations co-occur and thus there is potential for gene flow to counteract maintenance of coordinated seasonal adaptations (i.e., early timing and cold tolerant alleles in cold environments). Natural selection might be independently strong enough to maintain appropriate allelic combinations and promote coordination, however the genomic architecture of these traits will also be important. If putative loci underlying cold tolerance in the ECB moth map to a known ~ 10 Mb non-recombining region of the Z chromosome (a putative inversion) [39, 40], cold hardiness and early timing would be expected to be broadly correlated in field populations, perhaps even where hybridization rates reach 5–10% [21, 54]. Double recombination and gene conversion can eventually break co-adapted gene complexes within rearrangements [55]. However, strong evidence suggests the circadian clock gene *period* controls early or late diapause termination timing in the ECB moth [34, 46], and in *D. melanogaster*, splice variants in the same gene enhance cold tolerance (chill coma recovery) [56]. If *period* has direct effects on seasonal timing and seasonal tolerance through pleiotropy, then widespread and long-term maintenance of co-adapted seasonal traits will be expected.

Multiple physiological mechanisms could account for enhanced thermal tolerance. Insects are known to survive extreme cold through changes in membrane fluidity or cytoskeletal organization, altered regulation of heat shock proteins affecting the rate of refolding of temperature-denatured proteins, and change in the amount of polyhydric alcohols or antifreeze proteins sequestered in tissues to avoid freezing [21]. Prior work has focused on freeze avoidance mediated by accumulations of glycerol as the primary overwintering strategy used by *Ostrinia* [38–40]. However, the lower thermal limit in E strain insects appears unrelated to changes in freeze avoidance because no significant differences in supercooling point were found between univoltine Z and bivoltine E populations across life stages (Fig. 6). Hence, some other mechanism besides glycerol production must be accounting for variation in temperature tolerance in this system. Interestingly, though strains had a similar cold-tolerance capacity in the diapausing life stage (LT50: − 20.04 °C univoltine Z vs. -20.08 °C bivoltine E), overall the E strain did survive better across low temperature treatments (Fig. 5c). As bivoltine E and univoltine Z larvae share the same low-temperature habitat during diapause, enhanced thermal tolerance could possibly stem from a generalized mechanism that is “turned on” across all E strain life stages.

## Conclusions

Ecological speciation by allochrony is predicted to result in temporally isolated populations that are locally adapted to distinct temporal environments, but temporal specialization or “adaptation by time” remains an understudied facet of ecological speciation theory [21, 54, 57–59]. Our results suggest that isolation by time and adaptation by time can both contribute to population divergence, and they underscore a need for greater understanding of cyclical selective pressures that are inherent features of seasonal habitats. Natural selection across seasonal environments may promote local temperature adaptation as it has in ECB moths, *Rhagoletis* flies [47], and pine processionary moths [13], however diverse selective agents in addition to temperature (predation, competition, precipitation, etc.) fluctuate across each year. Thus, a multitude of temporally variable conditions may be crucial, yet underappreciated, drivers of biological diversity.

## Methods

### Estimating the phenology of life stages and their thermal environments

The ECB life cycle consists of egg, larva (5 instars), pupa, and adult stages. Larvae chew tunnels into host plants and both larval and pupal stages occur within host plant tissues. The 5th instar larva is the overwintering, diapausing life stage. We estimated life-stage specific phenology of univoltine Z and bivoltine E populations using phenology data for adult mating flights collected by the New York State Cooperative Extension Service. The Sweet Corn Pheromone Trapping Network (sweetcorn.nysipm.cornell.edu) monitors adult flights at sites throughout NY from May to October. For at least 1 year during a 12-year period (1999–2010), cooperating farms (*n* = 41) deployed two Scentry *Heliothis* traps (Scentry Biologicals, Inc., Billings, MT) at the edges of corn fields. Each trap was baited with a lure that released a synthetic pheromone that mimics the female blend produced by the E strain (99:1 E/Z 11–14:OAc) or the Z strain (3:97 E/Z 11–14:OAc) (Trécé, Inc., Adair, OK). Each week, traps were emptied of males and lures were replaced.

Most NY sites trapped both E and Z moths, but only four showed high densities of bivoltine E and univoltine Z moths (*n* > 100) in at least 1 year [27]. Of these, Farmington, NY (42.98775°N, − 77.30946°W) had trapping data for all 12 years of the monitoring period (Supplementary Table 2) and therefore it was selected to estimate phenology of bivoltine E and univoltine Z life cycles.

Each year, flight periods were defined as at least two-week intervals in which five or more males were caught in pheromone traps. The number of degree days (DD) required for development was then used to make weekly estimates of the phenology of other life stages. Previous studies suggest that the required number of degree days for development of corn borers is similar for populations across the United States [60]. Therefore, a single developmental degree-day model was used to predict phenology of both bivoltine E and univoltine Z moths: 212 accumulated degree days between adults and 1st instar larvae, 580 degree days between 1st instar larvae and 5th instar larvae, 210 degree days between 5th instar larvae and pupae, and 190 degree days between pupae and adults (Supplementary Figure 1 [61];). To back-calculate the dates in which post-winter pupal stages occurred, 190 degree days were subtracted from adult flights. Daily climate data from 1999 to 2010 were mined from the National Oceanic and Atmospheric Administration’s (NOAA) National Weather Service database (http://w2.weather.gov/climate/). The closest weather station with historical climate data was in Geneva, NY, ~ 27 km away from Farmington, NY. Data included high temperatures, low temperatures, and climate normals. Daily heat units were estimated by subtracting the minimum base temperature for corn borer development (10 °C) from the mean daily temperature [60–62]. This calculation excludes days in which the mean temperature was less than the minimum temperature required for development (< 10 °C). After predicting life cycle timing for each strain by year, the mean and standard error was calculated for the predicted start and end dates of each life stage across the entire 12-year monitoring period.

Given life stage specific predictions of phenology for bivoltine E and univoltine Z moths, we estimated seasonal fluctuations in temperature experienced by univoltine Z and bivoltine E populations. For time intervals in which each life stage for each strain was predicted to occur, climate data were used to characterize the daily high and low in temperature.

### Cold-tolerance

We next addressed how phenological shifts and exposure to different thermal environments might relate to thermal tolerance. To help motivate a broader analysis of populations, measurements were made from a well-characterized population pair of bivoltine E and univoltine Z moths from central NY that have been maintained in the laboratory [17, 27, 30, 34, 45]. Both strains have equivalent generation times of ~ 30 days in conditions that support direct development. These populations have had their genomes sequenced and are known to show fixed differences at loci underlying pheromone communication (*pgFAR*) and diapause timing [46]. Lethal temperatures (LT) and supercooling points were measured for the direct developing life stage that follows diapause (pupa), a representative direct developing life stage preceding diapause (non-diapausing 5th instar larva), and the cold-acclimated diapausing life stage (diapausing 5th instar larva). Diapausing larval stages of bivoltine E and univoltine Z populations both experience the same harsh winter environment, thus we hypothesized a lack of divergence in cold hardiness during this life stage. The thermally vulnerable direct developing larval and pupal life-stages immediately preceding and following diapause were selected to test adaptation by time, as displacement in time may shift these stages into distinct selective environments between the strains (Fig. 1). To test for variation in cold tolerance, lethal temperatures were compared across populations and life stages. Supercooling point (SCP), or the temperature at which corn borers spontaneously freeze, was also measured to assess the possible physiological mechanism of variation in cold tolerance.

#### Corn borer rearing

Corn borers were acquired from the field at locations where univoltine Z and bivoltine E populations co-occur – > 500 males and > 500 females of each strain were collected from Bouckville, NY (42.8892°N, 75.5513°W) in 1994 and Geneva, NY (42.8680°N, 76.9856°W) in 1996, respectively. There are no significant differences in monthly temperature between these sites (Supplementary Table 1). At both sites in the early spring, fifth instar diapausing larvae or pupae were removed directly from sweet corn (*Zea mays*) stubble, which is the bottom 30–45 cm of the dead stalk that remains after the previous year’s harvest. Breeding colonies were reared under benign common-garden conditions and kept under constant temperature (26 °C) and a “long-day” photoperiod (16:8 LD), thereby minimizing selection on temperature sensitivity. Larvae were fed a standard artificial European corn borer diet (Southland Products, Lake Village, AR, USA). The effects of genetic drift were minimized by maintaining breeding colonies for each strain *en masse* (> 100 breeding pairs per generation).

Direct development and diapause can be induced in both strains of corn borers by long-days (16:8 LD) and short-days (12:12 LD), respectively [17, 30, 34, 43]. Direct developing 5th instar larval and pupal life stages were obtained by rearing corn borers from eggs under 16:8 LD photoperiod at 26 °C. For both strains, 5th instar larvae were collected after 24 days in these conditions, and pupae were collected 3–4 days after pupation. The 5th instar was chosen as a representative direct developing larval stage, as it is the easiest larval instar to stage due to behavioral changes (e.g., cessation of feeding and migration to the top of the rearing container).

To induce diapause, 1st instar larvae were transferred just after hatching to 12:12 LD and 23 °C. To simulate winter conditions and cold acclimatize diapausing larvae, 5th instar larvae were transferred on day 24 to 12:12 LD with a cycling thermoperiod consisting of 10 °C during photophase and 0 °C during scotophase. Cold-acclimation lasted 20 days. To confirm that both direct developing and diapausing larval groups had reached the 5th instar under the experimental conditions, head-capsule width was measured across a subset of individuals for each strain (*n* = 32 per strain per larval life stage) [63].

For all experimental treatments, larvae were held overnight in individual plastic cups with a moist piece of dental wicking but without food to facilitate purging of the gut of any ice-nucleators, which have known impacts on cold-hardiness measures in corn borers [38]. Pupae were also held in individual plastic cups with a moist piece of dental wicking overnight. After the holding period, residual moisture, which can influence survival at subzero temperatures [38], was removed from the surface of each individual by blotting with tissue before experimental treatments.

#### Lethal temperature

Corn borers were placed into individual gelatin capsules (21 mm × 7 mm) and randomly assigned to a treatment consisting of a 2-h exposure of sub-zero temperature. Temperature treatments were divided into the following temperature categories: from − 2 °C to − 16 °C at 2 °C intervals for non-diapause 5th instar larvae (*n* = 4 groups of 8–10 individuals per condition per strain), from − 2 °C to − 16 °C at 2 °C intervals for pupae (*n* = 4 groups of 8–10 individuals per condition per strain), and from − 4 °C to − 24 °C at 4 °C intervals for cold-acclimated diapause 5th instar larvae (*n* = 3 groups of 8–10 individuals per condition per strain). Total sample sizes for treatments ranged from 192 to 327 individuals (Table 1). A different thermal range was tested in cold-acclimated diapause groups due to an expected lower lethal temperature for diapause larvae [38–40, 64–66].

Each treatment group was placed into individual 50 mL conical tubes, which were submerged into a Neslab RTE-140 M refrigerated bath circulator (Thermo Neslab, Waltham, MA, USA) containing a 1:1 solution of propylene glycol and water (Sigma-Aldrich, St. Louis, MO, USA). The bath was pre-cooled prior to submersion of the treatment groups, thus resulting in a step transfer, or rapid transfer of corn borers from permissive growing temperatures to cold. Control groups had no treatment and were maintained under the rearing conditions for each life stage during the treatment period.

After the cold exposure treatment, corn borers were transferred to individual cups with a moist piece of dental wicking and returned to favorable growing conditions at 26 °C in 16:8 LD. Diapausing groups were also moved to 26 °C in 16:8 LD. After 10 days, borers were scored for mortality. For larval groups, death was clearly evident with larvae becoming non-mobile, black, and shriveled [38]. In the pupal groups, death was also clearly evident with pupae becoming black and shriveled, or with adults failing to emerge completely from their puparium and perishing.

#### Supercooling point

Increasing the levels of glycerol or other polyhydric alcohols is a common mechanism for insects to enhance their cold-hardiness by decreasing the temperature at which tissues freeze [36, 37], and corn borers are known to enhance their survival via this mechanism during overwintering diapause [40]. Thus, SCP was used to evaluate how altered regulation of glycerol might vary among strains and life stages. Individuals were placed into separate capsules with a type T copper constant thermocouple (sensitivity rating of ±0.2 °C) (Thermoworks, Lindon, UT, USA) fed into the interior. Each capsule was then submerged within a 50 mL conical tube in the refrigerated bath circulator. From 10 °C, the bath was cooled at a rate of − 1 °C per minute. Real-time temperature measurements were taken using a Picotech TC-08 datalogger and the PicoLog software (Pico Technology, St Neots, Cambridgeshire, UK) for eight samples simultaneously. Following prior studies [40, 67], the lowest temperature reached before a spike in temperature that results from the exothermic nature of ice crystallization was recorded as the SCP.

#### Statistical tests

All analyses were conducted in R v.3.1.1 [68]. For cold-shock experiments, in order to understand the temperature-specific effects on death due to treatments, mortality was normalized to the control groups using the Henderson-Tilton formula [69]:

$$ \% Mortality=\left(1-\frac{Ta\ast Cb}{Ca\ast Tb}\right)\ast 100 $$

where *Cb* is the number of individuals in the control group before treatment, *Ta* is the number of individuals in the treatment group after treatment, *Ca* is the number of individuals in the control after treatment, and *Tb* is the number of individuals in the treatment group before treatment. After normalization, we implemented a hard cap at 0% mortality and 100% survival for treatment groups with better survival than the controls. Resultant normalized count data were rounded to the nearest whole number. Control mortality was < 15%.

An analysis was run to determine the temperature at which 50% of the population perished (LT50) for all life stages and both strains. Temperature doses were transformed to positive values by adding 25 (where the temperature range from − 24 to − 2 was transformed to 1 to 23) and a two-parameter log-logistic function was then fit to the data using the drc package v.2.5–12 [70] for each strain by life stage:

$$ f(x)=\frac{1}{1+\exp \left(b\left(\log (x)-\log (e)\right)\right)} $$

where *b* is the slope, *e* is the inflection point (LT50), and the upper and lower limits are fixed at 1 and 0 respectively. 95% confidence intervals were estimated using the delta method. Finally, significance between LT50s of groups was evaluated by comparing the ratio of LT50s between populations or life stages [68], followed by a correction for multiple testing [71].

In order to determine the effects of temperature dose, strain, and life stage on mortality, multiple generalized linear models (GLMs) were fit to the data. The first GLM investigated the non-diapausing pupal and larval life stages. A model was fit using the statistics package *glm* function v.3.2.2 [68] with temperature dose, strain, and life stage as factors. Temperature dose was considered a discrete factor within these models. Interaction terms with no significant effects were removed to simplify the model. This was followed by Tukey-adjusted post-hoc pairwise comparisons (Tukey’s honest significant difference test (Tukey’s HSD)) of the least-squares mean survival of each strain and life stage combination, nested within temperature using the *lsmeans* function v.2.20–23 [72].

Due to the different temperature sampling design used for cold-acclimated diapausing larval groups, a second GLM was fit for these groups with temperature dose and strain as factors, and again, we conducted Tukey-adjusted post-hoc comparisons of the strains nested within temperature. In order to determine if cold-acclimated diapause groups were significantly different from non-diapausing larvae at the lowest common temperature condition measured (− 16 °C), a Fisher’s Exact test [73] was performed on normalized dead and alive counts within each strain.

For SCP experiments, we tested for significant differences in SCP between life stages and strains. An analysis of covariance (ANCOVA) with mass as a covariate followed post-hoc by Tukey’s HSD test was run using the statistics package *aov* function v.3.2.2 [68].

## Supplementary information

**Additional file 1.** Supplementary Figure 1. Degree-day requirements for life stages of the European corn borer moth. The number of Celsius degree days between the timing of adult male capture in pheromone traps (T_0_) and other life stages [61]. Arrows indicate addition or subtraction of degree days from T_0_.

**Additional file 2. **Supplementary Figure 2. Maximum daily temperatures reported from 1999 to 2010 for the predicted temporal niches of (a) E and Z strain pupae (Wilcox Rank Sum, W = 154,390, *p* < 0.0001), and (b) E strain second-generation and Z strain single-generation first through fourth instar larvae (Wilcox Rank Sum, W = 283,840, *p* < 0.0001).

**Additional file 3. **Supplementary Figure 3. Variation in supercooling point. Z (red) and E (blue) corn borer strains by life stage. Bivoltine E cold-acclimated diapausing larvae had a significantly higher SCP than bivoltine E direct developing larvae (Tukey’s HSD, *p* = 0.01), bivoltine E pupae (Tukey’s HSD, *p* < 0.001), and univoltine Z pupae (Tukey’s HSD, *p* < 0.001).

**Additional file 4.** Supplementary Table 1. Results from glm testing the effects of collection location and minimum monthly temperature from 2008 to 2010.

**Additional file 5.** Supplementary Table 2. Number of E and Z adult male corn borers collected from Farmington, NY between 1999 and 2010.

## Data Availability

The datasets generated and analyzed during the current study are available in the Dryad repository, https://datadryad.org/stash/share/7JPCw1kwr7rA07oyRahGfJMxKzFvPu7TQ_ZbfB6q3NA.

## Abbreviations

DD: Degree-day
ECB: European corn borer moth
L:D: Light:dark
LT: Lethal temperature
SCP: Supercooling point
