# Supplementary figures and images for: Phenology-dependent cold exposure and thermal performance of Ostrinia nubilalis ecotypes
Source: BMC Evol Biol. 2020 Mar 6;20:34. doi: 10.1186/s12862-020-1598-6 (PMC7059338; doi:10.1186/s12862-020-1598-6)

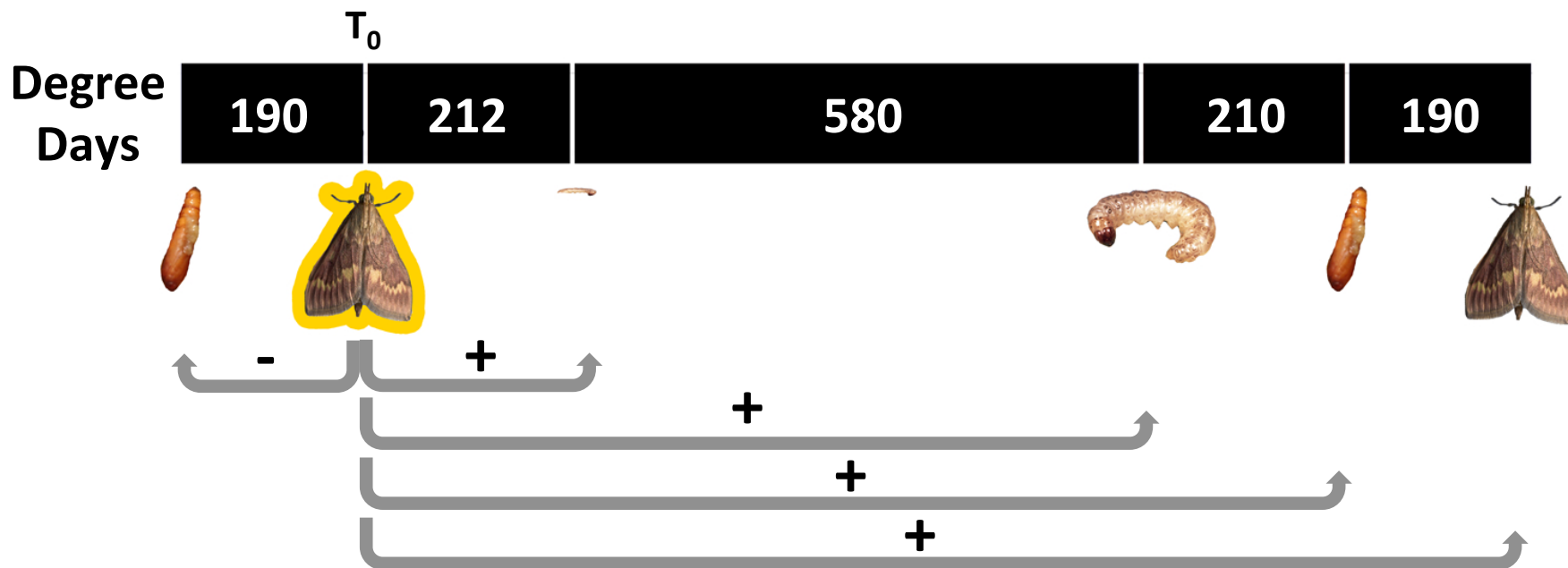

Supplement: Supplementary file 1 — Additional file 1. Supplementary Figure 1. Degree-day requirements for life stages of the European corn borer moth. The number of Celsius degree days between the timing of adult male capture in pheromone traps (T0) and other life stages [61]. Arrows indicate addition or subtraction of degree days from T0. [file 12862_2020_1598_MOESM1_ESM.pdf]

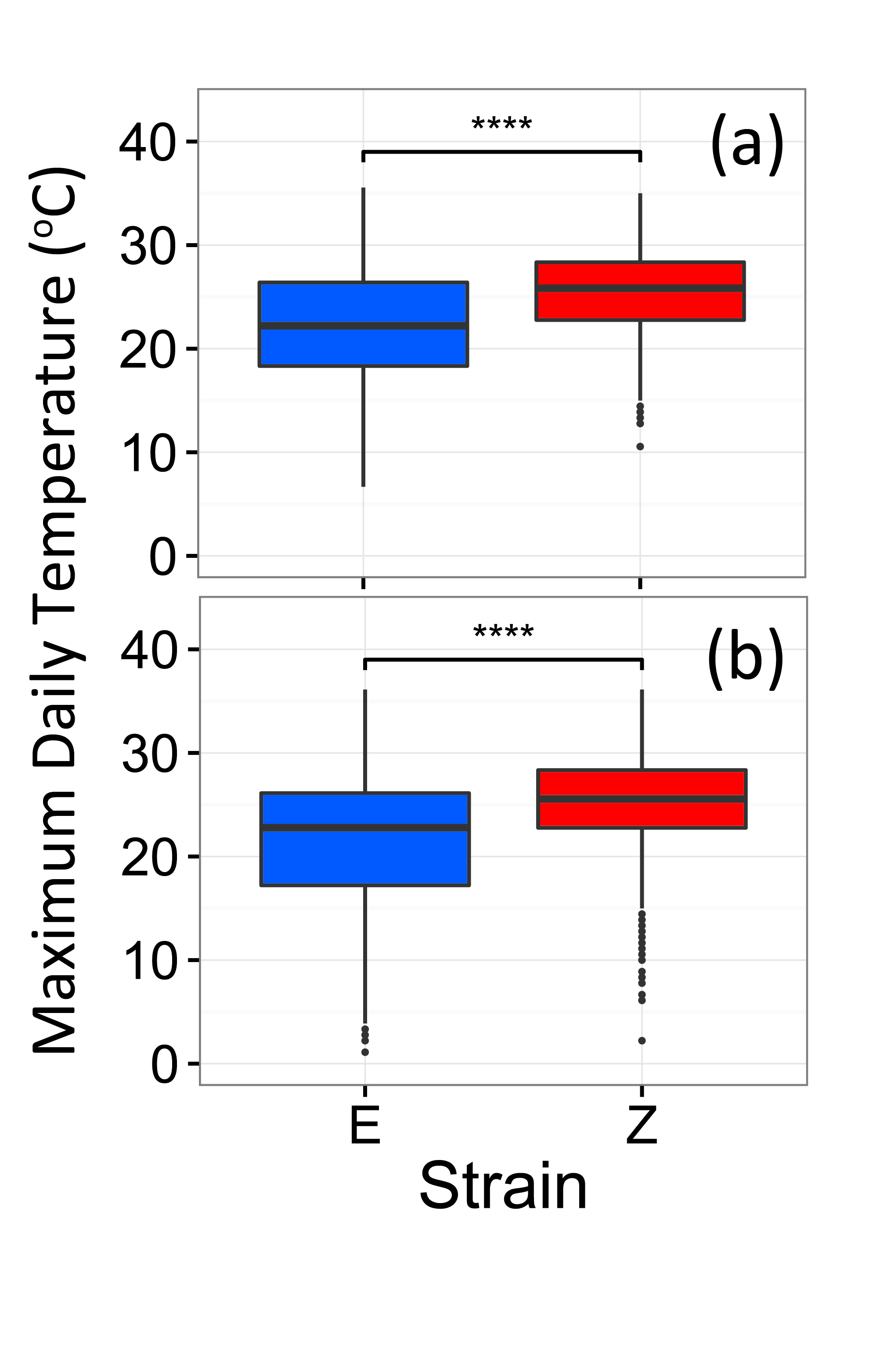

Supplement: Supplementary file 2 — Additional file 2. Supplementary Figure 2. Maximum daily temperatures reported from 1999 to 2010 for the predicted temporal niches of (a) E and Z strain pupae (Wilcox Rank Sum, W = 154,390, p < 0.0001), and (b) E strain second-generation and Z strain single-generation first through fourth instar larvae (Wilcox Rank Sum, W = 283,840, p < 0.0001). [file 12862_2020_1598_MOESM2_ESM.jpg]

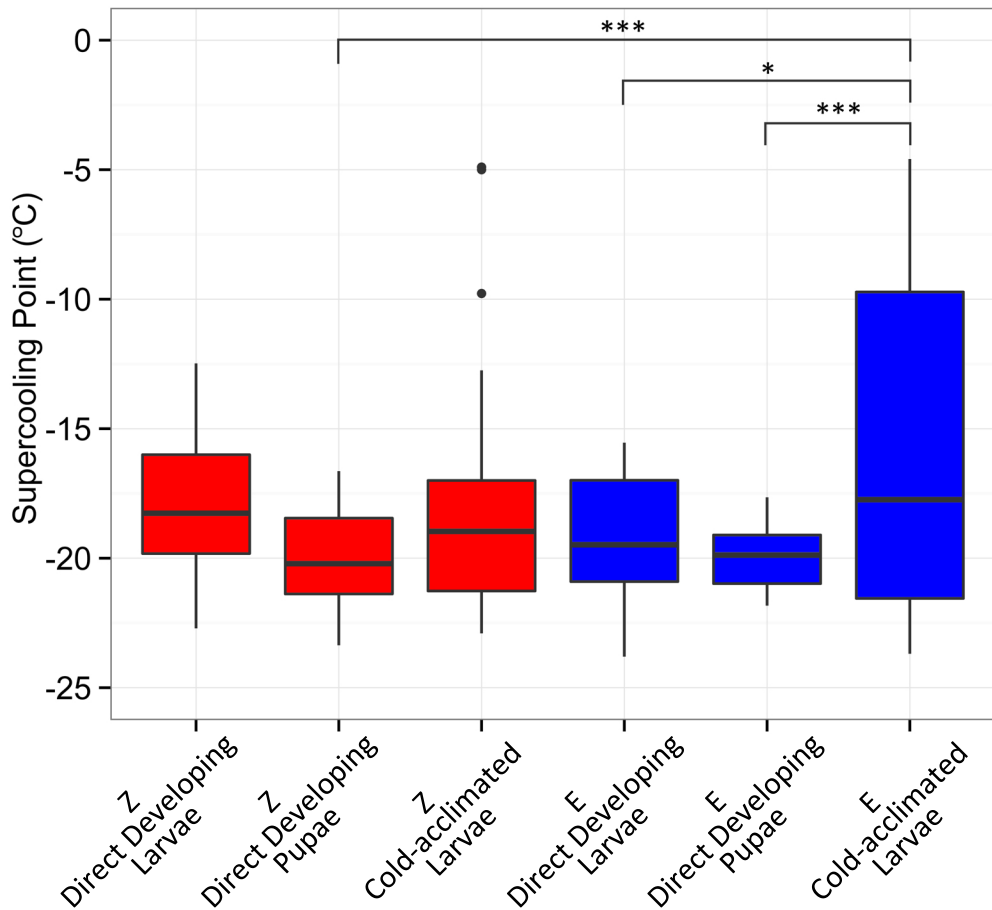

Supplement: Supplementary file 3 — Additional file 3. Supplementary Figure 3. Variation in supercooling point. Z (red) and E (blue) corn borer strains by life stage. Bivoltine E cold-acclimated diapausing larvae had a significantly higher SCP than bivoltine E direct developing larvae (Tukey’s HSD, p = 0.01), bivoltine E pupae (Tukey’s HSD, p < 0.001), and univoltine Z pupae (Tukey’s HSD, p < 0.001). [file 12862_2020_1598_MOESM3_ESM.pdf]
